# Cascaded Degradation-Aware Blind Super-Resolution

**DOI:** 10.3390/s23115338

**Published:** 2023-06-05

**Authors:** Ding Zhang, Ni Tang, Dongxiao Zhang, Yanyun Qu

**Affiliations:** 1 School of Information, Xiamen University, Xiamen 361005, China; 2 School of Science, Jimei University, Xiamen 361021, China; 202111701018@jmu.edu.cn (N.T.); zdx1980@jmu.edu.cn (D.Z.)

**Keywords:** image super-resolution, multiple degradation factors, blur kernel estimation, contrast learning

## Abstract

Image super-resolution (SR) usually synthesizes degraded low-resolution images with a predefined degradation model for training. Existing SR methods inevitably perform poorly when the true degradation does not follow the predefined degradation, especially in the case of the real world. To tackle this robustness issue, we propose a cascaded degradation-aware blind super-resolution network (CDASRN), which not only eliminates the influence of noise on blur kernel estimation but also can estimate the spatially varying blur kernel. With the addition of contrastive learning, our CDASRN can further distinguish the differences between local blur kernels, greatly improving its practicality. Experiments in various settings show that CDASRN outperforms state-of-the-art methods on both heavily degraded synthetic datasets and real-world datasets.

## 1. Introduction

Single-image super-resolution (SISR) aims to reconstruct high-resolution (HR) images from low-resolution (LR) images [1], which is an important research direction in the field of computer vision. Since Dong et al. [2] first used a convolutional neural network (CNN) for the image super-resolution reconstruction task (SRCNN), and the performance of SISR was significantly improved. On this basis, researchers have proposed various better SR networks. For example, Kim et al. [3] proposed a very deep convolutional network for super-resolution (VDSR) that uses the residual network [4] and gradient clipping technology to effectively improve the accuracy of the model while reducing the model parameters. These methods usually assume that the image degradation process is known, and all default to bicubic downsampling. However, the degradation process of real images is often more complex. Therefore, the real degradation process may not match the training data [5], resulting in a significant difference in the performance of the model when the above SR method is used to process the real image degradation [6] and produce artifacts [7].

To make SR methods applicable to real images, researchers have proposed a series of non-blind super-resolution (non-Blind SR) and blind super-resolution (Blind SR) methods. The non-Blind SR method takes the real degradation information as an additional input to establish the connection between HR and LR images. The non-Blind SR method has produced an excellent performance, but it is frequently challenging to obtain the real degradation information of the image; thus, its applicability in real-world situations is obviously constrained.

The Blind SR method can effectively break through the limitations of non-Blind SR. Currently, the Blind SR method estimates the blur kernel before reconstructing the LR image [8]. A majority of current blur kernel estimate networks, however, make the assumption that the image’s blur kernels are distributed uniformly in space. The blur kernels at different positions of the image will be different due to the influence of different environmental factors, such as object motion, depth difference, and non-ideal imaging factors (e.g., out of focus or camera shake [9,10]). In addition, real images contain noises. If the blur kernel is estimated directly, the estimated blur kernel will deviate from the real blur, resulting in poor reconstruction results.

In this paper, we propose a Cascaded Degradation-Aware Super-Resolution Blind Network (CDASRN), which introduces a noise estimation subnetwork before performing blur kernel estimation, thereby eliminating the influence of noise on blur kernel estimation. Subsequently, we added a contrast enhancement module to the network to improve the network’s ability to identify local features. The method proposed in this work outperforms other state-of-the-art methods in the test set in terms of quantitative and qualitative results, taking into account multiple image degradation characteristics and uneven spatial distribution of blur kernels. The main contributions of the study are summarized as follows:

- We designed a spatially varying blur kernel estimation network to estimate the blur kernel corresponding to each pixel of the LR input image and used the contrastive learning method for further enhancement.
- We introduced a noise estimation subnetwork to eliminate the influence of noise on blur kernel estimation, thus improving the accuracy of blur kernel estimation.
- Extensive experiments show that the proposed CDASRN can achieve an excellent Blind SR performance in different degradation settings, from simple to complex.

## 2. Related Work

In this section, we briefly review SISR and related methods, including contrastive learning.

### 2.1. Non-Blind Super-Resolution Reconstruction

Since the pioneering work of SRCNN [2], which used a three-layer convolutional network to learn image SR, most of the subsequent works have focused on optimizing network architectures [3,4,11,12,13,14,15] and loss functions [16,17,18]. The global residual learning module in VDSR [3] and the local residual learning module in SRRestNet [17] have effectively improved the model accuracy while reducing the model parameters. Dai et al. [14] designed a second-order attention mechanism based on SENet [15] and introduced a non-local neural network to learn local feature representations of images. HAN [19] introduces a hierarchical attention module to consider the correlation between multiscale layers. These CNN-based methods achieve a remarkable quantitative or qualitative performance on SISR with a single degradation factor. However, if the degradation in test images is different from that of a single degradation factor, they tend to produce over-sharpened or blurred results [20].

When the degradation information of the real image is known, Zhang et al. [8] proposes a dimension expansion strategy that allows the convolutional super-resolution network to take the blur kernel and noise as additional input, which improves the performance. Xu et al. [9] also used the same degradation information fusion strategy but added dynamic upsampling filtering to their backbone network to further enhance the SR performance. However, these methods require accurate degradation information, which is not available when the degradation factors are unknown.

### 2.2. Blind Super-Resolution Reconstruction

Unlike the non-Blind SR method, Blind SR methods only require LR images as input and thus are not required to offer precise degradation information. KernelGAN, proposed by Bell-Kligler et al. [10], is a pioneering work that introduced a deep linear network for SR kernel estimation. It demonstrated that the network could successfully estimate the SR kernel, but it fails to fully exploit the self-similarity property that is fundamental to SISR kernel estimation. Thus, Park et al. [21] proposed total-variation-guided KernelGAN (TVG-kernelGAN), which efficiently utilizes self-similarity by weighting the input image. Gu et al. [22] proposed an iterative optimization scheme iterative kernel correction (IKC) that uses the estimated degradation parameters, such as the blur kernel, to assist image reconstruction and then uses the obtained reconstructed image to further estimate the degradation parameters. Through the iterative joint optimization of degradation estimation and image reconstruction, the estimated degradation parameters and the reconstructed HR image are more reliable, but multiple iterations are required, resulting in a large amount of calculation.

Different from the joint optimization method, Tao et al. [23] designed a spectrum-to-kernel (S2K) network, which transformed the blur kernel estimation from the spatial domain to the frequency domain, reducing the kernel estimation error. Combining S2K with existing non-Blind SR methods, the method achieves an excellent reconstruction performance. To address the model overfitting problem, El Helou et al. [24] propose a stochastic frequency masking of images used in training to regularize the network. To estimate the real blur kernel, Liang et al. [25] designed a spatially varying blur kernel estimation network with a suitable receptive field. Besides designing explicit feature extractors, some works have started to explore the extraction of implicit degradation information to aid the reconstruction task. For example, DASR [26] uses regression network to infer degradation information, and MM-RealSR [27] uses metric learning to measure different levels of degradation. In order to simultaneously extract the content and deteriorated features. Zhou et al. [28] used a lightweight block-based encoder and used unsupervised degradation prediction. However, the degradation information estimated by these methods is not accurate enough to provide a degradation representation to guide SR. Therefore, we propose an explicit degradation estimation method that can estimate the degradation information more efficiently.

### 2.3. Contrastive Learning

Contrastive learning aims at learning low-dimensional representations of data by contrasting similar and dissimilar samples. Contrastive learning methods based on deep learning have been widely used in high-level vision tasks, such as image classification [29,30] and image segmentation [31,32]. Recently, there were also some works that apply contrastive learning to blind image super-segmentation. For example, Wang et al. [33] proposed an unsupervised implicit degradation factor estimation method based on contrastive learning and used it to guide the image reconstruction process. Zhang et al. [34] used contrastive learning to extract the resolution-invariant features of the LR image and then restored the lost high-frequency details of the LR image by shortening the distance between the reconstructed image and the HR image. Different from existing methods, we incorporated contrastive learning into the reconstruction network to enhance the network’s ability to capture local content features.

## 3. Proposed Method

Real-world image deterioration is a sophisticated process that is affected by a wide range of factors. This paper considers the spatially varying blur kernel, noise, downsampling, and JPEG compression factors in order to improve the algorithm’s robustness to the real degradation process, and it assumes that the degradation process is formulated as follows:

(1) $$y={\left((x⊗K){↓}_{s}+n\right)}_{{\mathrm{JPEG}}_{q}},$$

where y and x represent the LR images and HR images, respectively; s represents the scaling factor; n represents additive white Gaussian noise; q represents JPEG compression factor; ⊗ represents convolution operation; K is the blur kernel matrix; and K’s the (i,j) position is the anisotropic Gaussian blur kernel at the (i,j) pixel in x. Compared with the isotropic Gaussian blur kernel, the anisotropic Gaussian kernel is common and can be regarded as a combination of motion blur and isotropic blur kernel. In addition, ${↓}_{s}$ represents the downsampling operation with a scaling factor of s, and most reconstruction algorithms use bicubic interpolation, making it impossible to deal with the complex degradation process of real images. In addition to the conventional bicubic downsampling ($D_{bicubic}^{s}$) and bilinear downsampling ($D_{bilinear}^{s}$), this paper uses a new downsampling strategy that is denoted as ($D_{down-up}^{s}=D_{down}^{s\cdot a}D_{up}^{a}$). It means that the downsampling with a reduction factor of s⋅a is performed, and then the upsampling with an amplification factor of a is performed, and the way of up/downsampling is randomly selected from bicubic sampling and bilinear sampling. Using this hybrid downsampling approach can introduce upscaling blur to LR images, which cannot be achieved with traditional downsampling operations.

**Figure 1:**
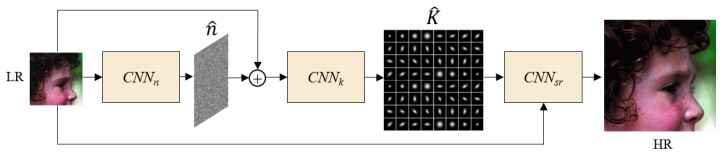
Overview of the CDASRN.

Because the majority of existing Blind SR methods only take into account the estimation of the spatially invariant blur kernel and ignore the noise caused by the decline in real image quality, the network’s performance while dealing with real images would decline significantly. To address the issues mentioned above, this paper proposes a novel network framework, CDASRN. We provide the overall framework of our method in Figure 1. It consists of three main components: the noise estimation subnetwork ($CNN_{n}$), the kernel estimation subnetwork ($CNN_{k}$), and the degradation-aware super-resolution subnetwork ($CNN_{sr}$). The input of the network is an LR image containing multiple degradation factors, and the degradation process is shown in Equation (1). The $CNN_{n}$ outputs the noise estimation feature map, $\hat{n}$, and is added with the original LR image as the input of $CNN_{k}$, which is used to estimate the spatially varying blur kernel, $\hat{K}$, of the LR image. This paper decreases the dimensionality of the blur kernel, stitches it, and then inputs it into $CNN_{sr}$ to generate the HR image because it is impractical to directly concatenate the acquired blur kernel with the LR image. In the following section, we introduce the details of our subnetwork.

### 3.1. Noise Estimation Subnetwork

While taking a picture, the image sensor’s field of vision is not bright enough, nor is the brightness uniform enough; therefore, noise is produced in the image. The predicted blur kernel will be erroneous if it is calculated directly on the LR image, which will have a negative impact on the quality of the reconstruction. This paper introduces a noise estimation subnetwork at the start of the network to minimize the impact of noise on later stages. The LR image with noise, blur, and JPEG compression is transmitted to the noise estimation subnetwork, and the output is a noise estimate feature map, $\hat{n}$. The noise estimation subnetwork is formulated as follows:

(2) $$\hat{n}=CNN_{n}(LR;\theta_{n}),$$

where $\theta_{n}$ represents the parameters in the noise estimation subnetwork.

**Figure 2:**
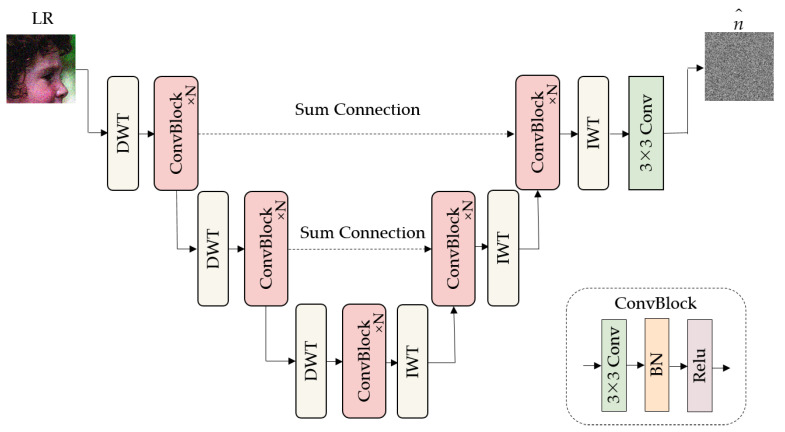
Architecture of the $CNN_{n}$.

For the structure of $CNN_{n}$, this paper adopts the 3-level MWCNN framework proposed by Liu et al. [35] which employs discrete wavelet transform (DWT) and inverse discrete wavelet transform (IWT) operations. The specific framework is shown in Figure 2. The network adopts the U-Net [36] structure, and the convolution block (ConvBlock) is used to extract features. Each convolution block is composed of a convolution, batch normalization, and activation layer. DWT captures the image details in four sub-bands: average (LL), vertical (HL), horizontal (LH), and diagonal (HH) information The spatial area of the sub-bands is four times smaller than the original image, and the transformed receptive field is doubled, so the framework saves computational cost while increasing the receptive field.

### 3.2. Kernel Estimation Subnetwork

Most existing Blind SR methods [8,9,22] assume that the blur kernel for each pixel is spatially invariant. However, this assumption does not match the reality that the blur kernel in real LR images varies across different regions. The image quality of the object captured by the imaging system will be unevenly blurred in multiple locations due to the sensor’s unequal jitter when recording the scene [37]. If the blur kernel of the whole image is assumed to be spatially invariant, it will not align with the actual blur kernel and will degrade the performance of SR methods. Moreover, the reconstructed images will be either too smooth or too sharp [22].

**Figure 3:**
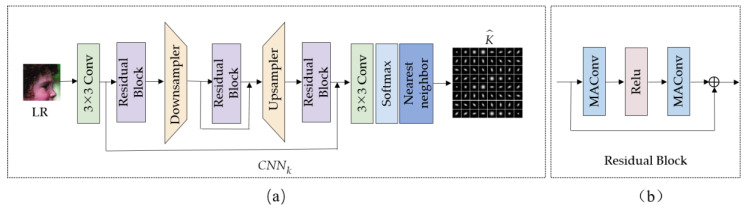
Architecture of the $CNN_{k}$. (**a**) The entire network framework of $CNN_{k}$, (**b**) The specific structure of Residual Block in $CNN_{k}$.

To address this issue, this paper takes into account the spatially variable blur kernel. Figure 3 depicts the detailed architecture of $CNN_{k}$, which consists of the kernel feature extraction and kernel reconstruction modules. Inspired by ESRGAN [38], the kernel feature extraction module consists of convolutional layers, residual blocks, skip connections, and up/downsampling, which adopts an encoder–decoder network architecture similar to U-Net [36]. To extract shallow image features, the LR image that has been fused with noise features is first fed into the convolutional layer. The shallow features are then fed into the residual block to extract kernel features. In order to use various levels of features and increase the expressiveness of the model, skip connections are added in the feature extraction module to each residual block unit, which is a convolutional layer-activation layer (ReLU)–convolutional-layer structure.

Liang et al. [25] argued that, for the task of spatially variable kernel estimation, it is necessary to maintain the locality of degradation. Therefore, they proposed a mutual affine layer (MAConv) that exploits the channel interdependence to enhance feature expressiveness without expanding the network’s receptive field. As a result, we used the MAConv module to replace the standard convolution in the residual block unit. Figure 3 displays the block structure that is Figure 3b. The model parameters and computation cost are decreased by around 30% when compared to the conventional convolutional layer.

Following feature extraction, the kernel reconstruction module uses convolutional layers and softmax layers to predict the blur kernel for each position in the LR map along the channel. The obtained blur kernel feature map is enlarged to the HR image size by using nearest-neighbor interpolation to produce the final blur kernel estimation results The kernel estimation subnetwork is formulated as follows:

(3) $$\hat{K}=R_{k}(F_{k}(LR,\hat{n};\theta_{k})),$$

where $\hat{K}$ is the kernel estimation result, and $\hat{K}\in R^{hw\times H\times W}$; H and W represent the length and width of the HR image, respectively; h and w represent the length and width of each blur kernel, respectively; $R_{k}$ represents the kernel reconstruction module; $F_{k}$ represents the kernel feature extraction module; and $\theta_{k}$ represents the parameters of the network.

### 3.3. Degradation-Aware Super-Resolution Subnetwork

**Figure 4:**
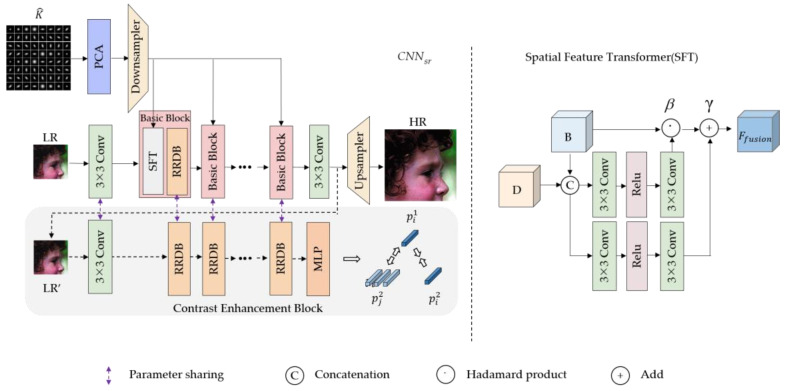
Architecture of the $CNN_{sr}$.

To fully utilize the obtained blur kernel information, we designed a degradation-aware super-resolution subnetwork based on contrastive learning, and the architecture is shown in Figure 4. The kernel estimation subnetwork extracted high-dimensional kernel information. If it is directly fed into $CNN_{sr}$, it will incur a large computation cost and a lot of data redundancy. Following the method of Zhang et al. [8], we used the principal component analysis (PCA) to reduce the dimensionality of the prediction blur kernel. The degradation-aware super-resolution subnetwork is formulated as follows:

(4) $$I^{SR}=CNN_{sr}(LR,\hat{K};\theta_{sr}),$$

where $I^{SR}$ is the result of the final super-resolution, and $\theta_{sr}$ is the parameter of $CNN_{sr}$.

To make the reconstruction network adaptable to different degradation processes, this paper reduces the dimensionality of the obtained blur kernel to form a degradation information matrix, D, and then continuously modulates the intermediate features in the cascaded basic blocks. The modulation aims to use the degradation information of the LR image to affect the intermediate features of the network, so that the SR reconstruction network can adapt to the image degradation and handle LR images under different degradation conditions. In Figure 4, the basic block (Basic Block) consists of two modules: the dynamic modulation layer (Spatial Feature Transformer, SFT) [39] and the residual block (Residual-in-Residual Dense Block, RRDB) [38]. SFT is primarily employed for dynamic feature modulation depending on degraded information, while RRDB is utilized for feature optimization to obtain features that are more conducive to HR image reconstruction.

SFT adopts the structure of 3×3 convolution—ReLU—3×3 convolution and learns the transformation matrix β and γ from the combination of feature map and kernel information. This process can be expressed as follows:

(5) $$F_{fusion}(B,{\hat{K}}_{pcas})=\beta ⊙B+\gamma ,$$

where $F_{fusion}$ represents the image after feature fusion, B is the original feature map, ${\hat{K}}_{pcas}$ represents the kernel feature compressed by PCA, β is the scale matrix, γ is the translation matrix, and ⊙ represents the Hadamard product.

We use the contrastive learning method to boost the network’s ability to gather local content by significantly distinguishing the blur kernel on each pixel. As shown in Figure 4, the image blocks extracted at the same spatial position of LR and LR’ are taken as positive samples, and the image blocks extracted at different spatial positions are taken as negative samples. The cascaded RRDB block is used to extract the features of LR and LR’, and then the feature vector is obtained through the multilayer perceptron network (MLP), denoted as $p^{1}$ and $p^{2}$, respectively. Therefore, the local block contrastive learning loss [30] can be expressed as follows:

(6) $$L_{LocCon}=\sum_{i=1}^{M\times N}-\log\frac{\exp(p_{i}^{1}\cdot p_{i}^{2}/\tau )}{\sum_{j\in Q}\exp(p_{i}^{1}\cdot p_{j}^{2}/\tau )},$$

where M and N represent the length and width of the LR image, respectively; Q represents the number of samples in the negative sample queue; $p_{j}^{2}$ represents the j-th negative sample; and τ is the temperature coefficient.

For the proposed Blind SR algorithm, the total loss includes the image reconstruction loss ($L_{SR}$), the blur kernel estimation network loss ($L_{K}$), the noise estimation network loss ($L_{N}$), and the local block comparison learning loss ($L_{LocCon}$). It is denoted as follows:

(7) $$L=L_{SR}+\lambda_{1}L_{K}+\lambda_{2}L_{N}+\lambda_{3}L_{LocCon},$$

where $\lambda_{1}$, $\lambda_{2}$, and $\lambda_{3}$ are the weight coefficients of $CNN_{k}$, $CNN_{n}$, and local block comparison learning loss, respectively. The CDASRN model is trained according to this overall learning objective.

## 4. Experiments

### 4.1. Experimental Settings

Datasets: In our experiments, we used the DIV2K [40] and Flickr2K [41] datasets together for training and testing. We selected 800 images on the DIV2K dataset and 2650 images on the Flick2K dataset as the sources of HR images x, which followed prior work [22]. Then, we generated a synthetic training set ${\left\{y_{i},x_{i},q_{i},s_{i},K_{i},n_{i}\right\}}_{i}^{N}$ with multiple degradation factors based on the model in Equation (1), where N denotes the total number of images in the dataset and i denotes the i-th images. We applied different combinations of compression $\left(q_{i}\in [50,100]\right)$, kernel width $\left(\sigma_{k}\in [0.7,10]\right)$ of blur kernel ($K_{i}$), and additive Gaussian noise level $\left(n_{i}\in [0,50]\right)$ to generate LR images (y). In addition, we randomly cropped HR and LR image patches from the corresponding LR-HR image pairs as training data, where the size of the HR image patch is 192×192. To enhance the training samples, pairs of image patches are randomly flipped by $90^{\circ }$.

To ensure the generalization of the model, Set5 [42], Set14 [43], and B100 [44] were chosen as the test sets. Set 5, Set 14, and B100 are datasets that contain 5, 14, and 100 images, respectively, of different scenes and objects from nature and artificial sources. We degraded these test datasets in different ways for various experiments to assess the performance of our model.

Experimental parameters: The network is optimized using the Adam [45] optimizer with a momentum of 0.9. The initial learning rate is set to $1\times 10^{-4}$, and is halved every 200 epoch of training, for a total of 800 epoch of training. Set the batch size to 16; the balance hyperparameter is set to $\lambda_{1}=1$, $\lambda_{2}=1$. For an isotropic spatially invariant Gaussian blur, the kernel is set to $\lambda_{3}=0$, and for a spatially varying Gaussian blur, the kernel is set to $\lambda_{3}=5\times 10^{-5}$. We trained and tested the model in this paper on the Tesla V100S-PCIE-32GB GPU and deployed it on the Pytorch framework.

### 4.2. Evaluation Metrics

We used the peak signal-to-noise ratio (PSNR) and structural similarity (SSIM) [46] as the evaluation metrics for our experiment. The PSNR measures the pixel-wise difference between the original and the reconstructed HR images by computing the peak signal-to-noise ratio. SSIM measures the structural similarity between the original and the reconstructed HR images by comparing their luminance, contrast, and correlation. We computed the PSNR and SSIM values on the Y channel of the YCrCb color space.

### 4.3. Experimental Process

**Figure 5:**
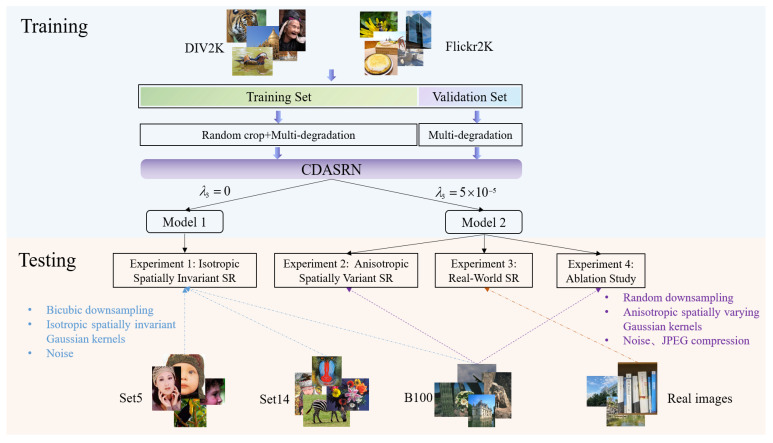
The overall framework of the experiment.

We designed four experiments to validate the effectiveness of our model, as shown in Figure 5. In the model training phase, to prevent overfitting, we used an early stopping technique to train our CDASRN network. We split Flickr2K and DIV2K into training and validation sets with a 9:1 ratio and used the validation set to monitor the performance of the model during training and applied early stopping when the validation loss stopped decreasing for 10 consecutive epochs. For the training set, we randomly cropped 192 × 192 patches from each whole image and applied multi-degradation operations on them, using Equation (1). For the validation set, we directly applied multi-degradation operations on the whole image, using Equation (1). Then, we inputted all processed images into the CDASRN network for model training.

Since most Blind SR methods only handle isotropic spatially invariant Gaussian kernels, we first trained an SR model for isotropic spatially invariant Gaussian kernels (Model 1) by setting $\lambda_{3}$ in Equation (7) to 0 during training, for a fair comparison. In Experiment 1, we compared the performance of Model 1 with existing Blind SR methods on synthetic test datasets. The test datasets were created by applying isotropic spatially invariant Gaussian blur, bicubic downsampling, and noise addition on Set5, Set14, and B100.

Next, to better simulate the complex degradation process of real images, we trained an SR model (Model 2) that can deal with anisotropic spatially varying blur kernels by setting $\lambda_{3}$ in Equation (7) to $5\times 10^{-5}$ during training. We evaluated Model 2 on both synthetic and real datasets and analyzed the contribution of each module of CDASRN. Experiment 2 tested Model 2 on a more challenging synthetic dataset, which was created by applying random downsampling, anisotropic spatially varying Gaussian blur, noise addition, compression, and other operations on B100. Experiment 3 is a visual comparison of Model 2 on the real dataset. Experiment 4 studies the effectiveness of each module of the proposed CDASRN model. We provide more details in Section 5.

## 5. Results and Analysis

### 5.1. Experiments on Isotropic Spatially Invariant SR

Since most Bind SR methods only deal with isotropic Gaussian kernels, we first compare the performance of our proposed method with existing Blind SR methods in the case of isotropic space-invariant Gaussian kernels. We use isotropic Gaussian kernels with different kernel widths, $\sigma_{k}\in \{1.3,2.6\}$, to degrade GT images on three benchmark datasets: Set5, Set14, and B100. We only use bicubic downsampling as the sole downsampling method and construct the corresponding ×2, ×3, and ×4 LR test sets to evaluate the model performance. To test the effectiveness of the model, we consider two noise levels $\left(\sigma_{n}=15,50\right)$ to simulate moderate and severe degradation cases.

We compare the proposed CDASRN method with the recent Blind SR method. The comparison results are shown in Table 1, and the best results are marked in bold. The methods for comparison include ZSSR [47], IKC [22], SFM [24], and DMSR [48]. Among them, ZSSR is a zero-shot learning method that trains the SR network by learning the relationship between the LR image and its downsampled image; IKC and SFM only assume that the LR image has blur degradation. DMSR considers both the noise and blur kernel effects and uses the extracted degradation information to guide the meta-restoration network. The “-vn” in Table 1 represents the model obtained by randomly adding noise to the training dataset and retraining. Among them, $\sigma_{n}$ is the noise level, and $\sigma_{k}$ is the kernel width.

The experimental results in Table 1 show that ZSSR cannot learn the internal distribution information of the image, and its performance drops significantly when noise increases from 15 to 50. Second, because the original IKC and SFM models were trained under specific noise settings, they perform poorly when the actual noise differs from the training noise. Adding random noise to the training set for IKC and SFM improves their performance, but not by much. The method reported in this paper adopts the strategy of first removing the noise effect and then estimating the blur kernel. DMSR obtained good results in varied kernel widths and different noise intensities. Compared to DMSR, our method performs better. In the case of an isotropic Gaussian kernel, it demonstrates that CDASRN has good generalization performance.

**Figure 6:**
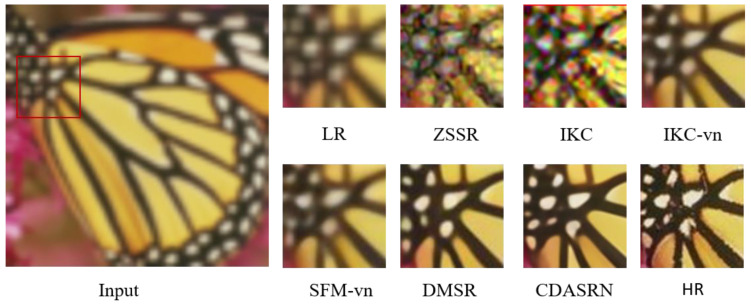
(×4) Reconstruction results of “butterfly” image in Set5 by different algorithms.

Figure 6 compares the qualitative of our method with several other methods in an isotropic spatially variant kernel setting. The input image is selected from the Set5 dataset, and an isotropic Gaussian kernel with a kernel width of 2.6 is used to degrade the real image, with a noise level of 50. The high noise level makes ZSSR produce noise results. The IKC approach has the same issue, and the reconstructed image quality is not much improved. The IKC-vn method, which retrains the dataset of the proposed degradation process, improves the visual restoration effect, but it is still blurry. Our method is closer to the HR image than DMSR because CDASRN restores details better, such as the texture of the butterfly, than DMSR does.

### 5.2. Experiments on Anisotropic Spatially Variant SR

To better simulate the degradation process of real images, we introduced various factors, such as spatially varying blur kernels, noise, JPEG compression, and random downsampling, into the degradation of the B100 dataset. To verify the effectiveness of our proposed method, we selected RCAN [14] and SRResNet [17] of the bicubic downsampling degradation method and tested their generalization ability to different complex degradation situations. For a fair comparison, we not only test their pre-trained models on the test set but also retrained RCAN and SRResNet on the same training set as ours and denoted them as ${\mathrm{RCAN}}_{r}$ and ${\mathrm{SRResNet}}_{r}$. Since the real degradation also involves factors such as blurring, downsampling, and noise interference, we compared the performance of some image restoration methods for denoising and deblurring which first denoise and deblur the image and then perform super-resolution reconstruction.

Three methods were constructed for comparison: (1) use the BM3D method proposed by Chen et al. [49] to remove noise and use the RCAN model (${\mathrm{RCAN}}_{r}\left(B\right)$); (2) after denoising with BM3D, use the dark channel prior BCP method proposed by Pan et al. [50] for blur kernel estimation and then use the SRMD model (SRMD(BB)); (3) first use the CBDNet [51] to estimate the real image noise, then use the Kernel-GAN to estimate the real image blur kernel, and finally use the SRMD model (SRMD(CK)). In addition, this paper also compares our model with existing Blind SR models: HAN [19], Kernel-GAN [10], IKC [22], and DASR [33]. HAN is an SR method for bicubic downsampling, while Kernel-GAN and IKC account for the blur kernel, and DASR is an unsupervised method for extracting degradation information. Table 2 lists the PSNR and SSIM results of the comparison method and the CDASRN method proposed in this paper, and the best results are marked in bold. In the table header $\left[s,\sigma_{n},q\right]$, s represents the super-resolution size, $\sigma_{n}$ represents the noise level, and q represents the compression factor of JPEG compression.

The SR model RCAN, which is based on bicubic downsampling training, performs worse when the downsampling factor is not bicubic downsampling. The PSNR of ${\mathrm{RCAN}}_{r}$ is better after retraining with multiple degraded datasets, but there is still a gap with CDASRN, demonstrating that the effectiveness of the method suggested in this research is not only due to the training of multiple deteriorated datasets. It also shows that the problem of the multi-degradation SR cannot be solved by simply stacking noise estimation, deblurring, and SR networks because the performance of ${\mathrm{RCAN}}_{r}\left(B\right)$ when using the combination of denoising network and SR network is still lower than that of ${\mathrm{RCAN}}_{r}$_._ Kernel-GAN designs an internal GAN framework based on patch dissimilarity, but its kernel estimation performance is limited, and it can only estimate a fixed blur kernel for an entire image, resulting in poor super-resolution results. IKC performs better than the aforementioned models since it can directly predict blur kernels from LR images. IKC uses a prediction and correction strategy; therefore, it starts by predicting the blur kernel, then applies image super-resolution, and last applies blur kernel correction. This method requires numerous iterations, resulting in time-consuming IKC throughout the testing stage. When the input image size is 192×192 and the magnification factor is 4, the time required for IKC is 15.6 s. The CDASRN model does not need to iterate multiple times, so it only takes 0.2 s to test an image. In complex degradation situations, CDASRN outperforms IKC and achieves the best objective metrics in less time.

**Figure 7:**
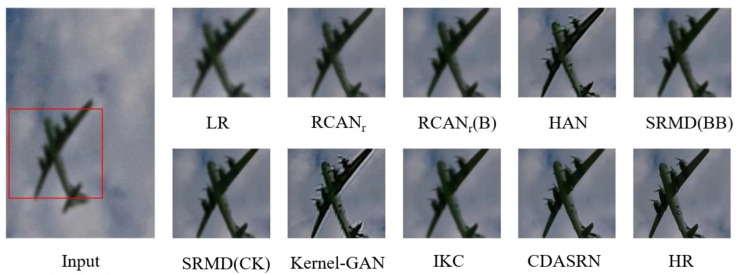
(×4) Reconstruction results of “img3096” image in B100 by different algorithms.

An example reconstruction image obtained using our method is shown in Figure 7. The input image is chosen from the benchmark test set B100, the spatially varying Gaussian kernel is randomly distributed, the noise intensity is 10, and the JPEG compression factor is 80. Even using the deblurring model BM3D, ${\mathrm{RCAN}}_{r}$ has a tendency to produce results that are too smooth. The HAN method, which assumes that the blur kernel is spatially invariant, is unable to estimate the blur kernel effectively, adding additional errors to the reconstructed image. SRMD(BB) improves image quality by using a denoising network, blur kernel estimate, and non-Blind SR processing, although it is still imperfect. The Kernel-GAN method’s reconstruction output is too sharp and produces a ringing effect. SRMD(CK) has solved the over-sharpening issue and produced better visual results; however, the image is too smooth with the IKC method. In comparison to the aforementioned techniques, CDASRN provides the most obvious reconstructed outcomes and the best texture detail restoration.

### 5.3. Experiments on Real-World SR

**Figure 8:**
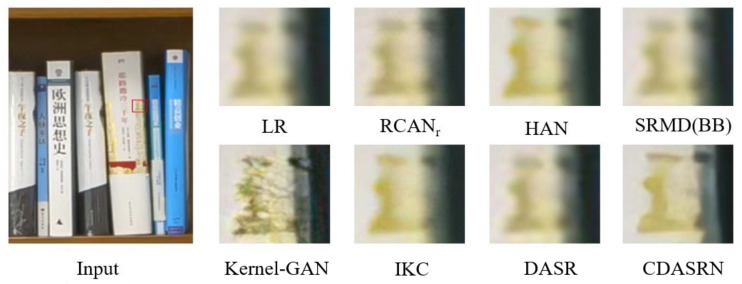
(×4) Reconstruction results of real images by different algorithms.

To evaluate and contrast how different methods perform in realistic scenarios, we present the reconstruction results of various methods on real images in Figure 8. CDASRN outperforms other methods in terms of the transparency with which detailed textures are recovered and the level of visual restoration, demonstrating definitely that the proposed method can be successfully applied to real situations after being trained on synthetic datasets.

### 5.4. Ablation Study

We conduct an ablation study to test under the degraded setting of the anisotropic blur kernel experiment to evaluate the efficacy of the proposed model network architecture and to confirm the effectiveness of each component of the proposed network. The network is designed in the five following ways: (1) Remove $CNN_{n}$ and $CNN_{k}$, remove the contrast enhancement module in $CNN_{sr}$, and increase the number of layers of basic blocks in $CNN_{sr}$ to ensure that the network has the same size as the original network, denoted as ${\mathrm{CDASRN}}_{\mathrm{sr}}$. (2) Maintain the whole CDASRN network model, but eliminate the noise and kernel estimation supervision loss during training, and only utilize $CNN_{sr}’s$ loss to supervise the entire network, denoted as ${\mathrm{CDASRN}}_{l}$. (3) Use $L_{K}$, $L_{N}$, and $L_{SR}$ to train $CNN_{n}$, $CNN_{k}$, and $CNN_{sr}$ separately and pass the LR images through the three subnetworks in the test phase to obtain the SR results. This network is recorded as ${\mathrm{CDASRN}}_{\mathrm{step}}$. (4) Remove the local position contrast learning loss used in $CNN_{sr}$ and record this network as ${\mathrm{CDASRN}}_{\mathrm{con}}$. (5) The network model proposed in this paper is recorded as ${\mathrm{CDASRN}}_{\mathrm{all}}$. Table 3 shows the quantitative results of the above network variants on the B100 dataset.

The ${\mathrm{CDASRN}}_{\mathrm{all}}$ proposed in this work produces the best PSNR/SSIM performance, as seen in Table 3. The end-to-end SR network is equivalent to the ${\mathrm{CDASRN}}_{\mathrm{sr}}$ in terms of network complexity. ${\mathrm{CDASRN}}_{\mathrm{sr}}$ is equivalent to simplifying the network into an end-to-end super-resolution network. Due to the lack of a kernel estimation subnetwork, the learning ability of the network variation blur kernel will be greatly weakened. ${\mathrm{CDASRN}}_{\mathrm{all}}$ has about 0.1~0.3 improvement in PSNR and about 0.01~0.03 improvement in SSIM compared to ${\mathrm{CDASRN}}_{\mathrm{step}}$ and ${\mathrm{CDASRN}}_{\mathrm{con}}$. The results validate the effectiveness of the network’s parts by demonstrating that the super-resolution of complex degraded images with uneven spatial distribution can be performed using the proposed network’s parts.

## 6. Conclusions

In this paper, we proposed a cascaded degraded-aware blind super-resolution reconstruction network. It combines the three main components of the noise estimation subnetwork, the kernel estimation subnetwork, and the degradation-aware super-resolution subnetwork Additionally, to further identify the blur kernels of various pixels and enhance the effect of reconstruction, this study took into account the Gaussian blur kernel with spatial variation for blur kernel estimation. The image super-resolution task of real sceneries was tackled in this paper using more complicated degradation modes. The extensive experimental results show that the method in this paper can achieve higher accuracy on benchmark datasets and is effective for reconstructing low-resolution images with multiple degrading factor noises. This study suggests a supervised blind super-resolution strategy that, in order to train the model, needs to know the true noise map and blur kernel. The image in a real scenario could suffer various degradations, making it impossible to obtain the noise map and blur kernel in their native format. As a result, dealing with numerous degraded real images using the supervised blind super-resolution approach is ineffective, and future research will focus on the unsupervised blind super-resolution method.

## Figures and Tables

**Table 1:** Comparison of objective parameters PSNR/SSIM of 2/3/4-times reconstruction results of different algorithms on data sets with different isotropic kernel widths.

Methods	$\left[\sigma_{k},\sigma_{n}\right]$	Set5			Set14			B100		
		×2	×3	×4	×2	×3	×4	×2	×3	×4
ZSSR	[1.3, 15]	25.33/0.5346	25.06/0.5478	24.28/0.5374	24.29/0.4891	23.95/0.4909	23.40/0.4833	24.06/0.4546	23.77/0.4554	23.28/0.4469
IKC		25.69/0.7128	25.77/0.6085	23.46/0.5038	24.25/0.6152	24.47/0.5400	21.82/0.4111	24.54/0.5827	24.26/0.4980	20.60/0.3343
SFM		26.15/0.6168	24.59/0.5233	21.64/0.4400	24.84/0.5523	23.53/0.4738	19.23/0.3243	24.78/0.5187	23.27/0.4337	19.39/0.2933
IKC-vn		30.56/0.8602	29.13/0.8278	28.26/0.8072	28.15/0.7654	26.97/0.7195	26.24/0.6881	27.34/0.7178	26.34/0.6705	25.65/0.6381
SFM-vn		30.66/0.8622	29.15/0.8304	28.00/0.8016	28.27/0.7717	27.11/0.7242	26.19/0.6885	27.45/0.7260	26.42/0.6780	25.64/0.6384
DMSR		31.13/0.8698	29.65/0.8403	28.48/0.8116	28.51/0.7735	27.24/0.7261	26.29/0.6895	27.54/0.7252	26.46/0.6738	25.70/0.6389
CDASRN		**31.19/0.8709**	**29.85/0.8484**	**28.80/0.8225**	**28.53/0.7757**	**27.62/0.7467**	**26.59/0.7108**	**27.60/0.7299**	**26.78/0.6966**	**25.92/0.6579**
ZSSR	[2.6, 15]	23.42/0.4378	23.40/0.4643	23.26/0.4826	22.53/0.3761	22.50/0.3989	22.43/0.4202	22.63/0.3436	22.64/0.3683	22.54/0.3888
IKC		24.33/0.6511	23.83/0.5079	21.60/0.3993	23.21/0.5606	22.85/0.4443	20.15/0.3058	23.71/0.5334	23.01/0.4111	19.58/0.2569
SFM		24.13/0.5255	23.17/0.4442	19.67/0.3246	23.07/0.4523	22.29/0.3820	18.27/0.2453	23.35/0.4225	22.28/0.3458	18.51/0.2192
IKC-vn		27.57/0.7849	26.52/0.7523	26.38/0.7503	25.52/0.6645	24.86/0.6373	24.79/0.6318	25.25/0.6182	24.74/0.5935	24.64/0.5875
SFM-vn		27.08/0.7686	26.80/0.7602	26.18/0.7425	25.38/0.6578	25.02/0.6423	24.71/0.6287	25.19/0.6141	24.94/0.6040	24.60/0.5855
DMSR		**28.81/0.8171**	**27.79/0.7943**	27.10/0.7743	**26.43/0.6916**	25.81/0.6672	25.30/0.6478	**25.86/0.6428**	25.33/0.6173	24.93/0.6005
CDASRN		28.63/0.8138	27.67/0.7921	**27.24/0.7777**	26.32/0.6888	**25.84/0.6721**	**25.37/0.6554**	25.81/0.6394	**25.37/0.6227**	**25.05/0.6077**
ZSSR	[1.3, 50]	17.12/0.1814	16.88/0.1906	16.71/0.2034	16.84/0.1641	16.65/0.1697	16.46/0.1764	16.70/0.1446	16.54/0.1483	16.40/0.1548
IKC		23.07/0.5762	17.20/0.2094	13.37/0.1298	22.46/0.4969	17.01/0.1880	12.52/0.0991	22.91/0.4709	16.68/0.1611	11.89/0.0758
SFM		18.59/0.2243	15.42/0.1665	11.88/0.0991	17.51/0.1817	15.24/0.1475	11.87/0.0857	17.56/0.1654	15.29/0.1307	11.59/0.0713
IKC-vn		27.27/0.7862	25.87/0.7466	24.81/0.7171	25.73/0.6766	24.65/0.6345	23.80/0.6071	25.24/0.6267	24.36/0.5883	23.67/0.5633
SFM-vn		27.32/0.7877	25.82/0.7472	24.78/0.7155	25.69/0.6762	24.64/0.6361	23.79/0.6074	25.23/0.6281	24.32/0.5904	23.66/0.5633
DMSR		**27.62**/0.7942	25.96/0.7533	24.98/0.7256	**25.86/0.6776**	24.76/0.6369	23.90/0.6088	25.30/0.6279	24.42/0.5897	23.71/0.5643
CDASRN		27.61/**0.7946**	**26.04/0.7572**	**25.14/0.7281**	25.82/0.6767	**24.78/0.6444**	**23.92/0.6131**	**25.32/0.6288**	**24.44/0.5970**	**23.84/0.5701**

**Table 2:** Comparison of objective parameters PSNR/SSIM of reconstruction results of different algorithms on anisotropic spatially varying Gaussian kernel blur B100 dataset.

Methods	[×2, 0, 80]	[×4, 10, 80]	[×4, 15, 50]
HAN	21.19/0.4243	20.65/0.3783	19.84/0.3451
RCAN	20.17/0.3875	19.54/0.3413	18.67/0.3321
${\mathrm{RCAN}}_{r}$	24.17/0.6675	22.13/0.5156	21.54/0.4913
${\mathrm{RCAN}}_{r}\left(B\right)$	23.13/0.6342	21.45/0.5942	21.21/0.4745
${\mathrm{SRResNet}}_{r}$	23.78/0.6523	21.32/0.4971	20.78/0.4531
SRMD(BB)	22.81/0.5581	22.21/0.5023	21.48/0.4813
SRMD(CK)	23.65/0.5673	22.43/0.5542	21.25/0.5721
Kernel GAN	23.49/0.6309	15.54/0.1032	15.11/0.1086
DASR	27.73/0.7681	24.96/0.6110	24.45/0.5891
IKC	27.81/0.7788	25.03/**0.6172**	24.56/0.5941
CDASRN	**28.30/0.7960**	**25.11**/0.6134	**24.89/0.6039**

**Table 3:** Comparison of objective parameters PSNR/SSIM of the reconstruction results of different network variant ablation experiments on t1he B100 dataset.

	${\mathrm{CDASRN}}_{\mathrm{sr}}$	${\mathrm{CDASRN}}_{l}$	${\mathrm{CDASRN}}_{\mathrm{step}}$	${\mathrm{CDASRN}}_{\mathrm{con}}$	${\mathrm{CDASRN}}_{\mathrm{all}}$
×2	24.16/0.6671	27.74/0.7731	27.71/0.7703	28.14/0.7902	**28.30/0.7960**
×4	22.16/0.5163	24.78/0.5827	24.97/0.5871	25.02/0.6091	**25.11/0.6134**

## Data Availability

No new data were created.
